# Cre/lox Studies Identify Resident Macrophages as the Major Source of Circulating Coagulation Factor XIII-A

**DOI:** 10.1161/ATVBAHA.117.309271

**Published:** 2017-07-26

**Authors:** Cora M.L. Beckers, Kingsley R. Simpson, Kathryn J. Griffin, Jane M. Brown, Lih T. Cheah, Kerrie A. Smith, Jean Vacher, Paul A. Cordell, Mark T. Kearney, Peter J. Grant, Richard J. Pease

**Affiliations:** From the Leeds Institute for Cardiovascular and Metabolic Medicine, LIGHT Laboratories, University of Leeds, United Kingdom (C.M.L.B., K.R.S., K.J.G., J.M.B., L.T.C., K.A.S., P.A.C., M.T.K., P.J.G., R.J.P.); and Clinical Research Institute of Montreal, McGill University, Canada (J.V.).

**Keywords:** animal models of human disease, bone marrow, platelets, macrophages, transplantation

## Abstract

**Objective—:**

To establish the cellular source of plasma factor (F)XIII-A.

**Approach and Results—:**

A novel mouse floxed for the *F13a1* gene, FXIII-A^flox/flox^ (Flox), was crossed with myeloid- and platelet-cre–expressing mice, and cellular FXIII-A mRNA expression and plasma and platelet FXIII-A levels were measured. The platelet factor 4-cre.Flox cross abolished platelet FXIII-A and reduced plasma FXIII-A to 23±3% (*P*<0.001). However, the effect of platelet factor 4-cre on plasma FXIII-A was exerted outside of the megakaryocyte lineage because plasma FXIII-A was not reduced in the Mpl^−/−^ mouse, despite marked thrombocytopenia. In support of this, platelet factor 4-cre depleted FXIII-A mRNA in brain, aorta, and heart of floxed mice, where FXIII-A^pos^ cells were identified as macrophages as they costained with CD163. In the integrin αM-cre.Flox and the double copy lysozyme 2-cre.cre.Flox crosses, plasma FXIII-A was reduced to, respectively, 75±5% (*P*=0.003) and 30±7% (*P*<0.001), with no change in FXIII-A content per platelet, further consistent with a macrophage origin of plasma FXIII-A. The change in plasma FXIII-A levels across the various mouse genotypes mirrored the change in FXIII-A mRNA expression in aorta. Bone marrow transplantation of FXIII-A^+/+^ bone marrow into FXIII-A^−/−^ mice both restored plasma FXIII-A to normal levels and replaced aortic and cardiac FXIII-A mRNA, while its transplantation into FXIII-A^+/+^ mice did not increase plasma FXIII-A levels, suggesting that a limited population of niches exists that support FXIII-A-releasing cells.

**Conclusions—:**

This work suggests that resident macrophages maintain plasma FXIII-A and exclude the platelet lineage as a major contributor.

## Abstract 2

Supplemental Digital Content is available in the text.

Factor (F)XIII-A is a moderately abundant plasma coagulation protein with a half-life of 5 to 10 days^1^; therefore, the cell type that releases FXIII-A into the plasma must be reasonably numerous. In mammals, FXIII-A is expressed in megakaryocytes, monocytes, osteocytes, chondrocytes, dendritic cells, and bone marrow (BM)–derived macrophages, which have polarized to the M2 phenotype.^2^ FXIII-A is expressed in resident macrophages in organs, including heart, aorta, skin, and placenta,^3–5^ but not in liver, spleen, and kidney.^6^ FXIII-A lacks a classical signal peptide,^7^ and its mechanism of release remains uncharacterized. As a consequence, plasma FXIII-A may not be released from every cell type in which it is expressed. The outcomes of human BM transplant (BMT) studies have variously implicated platelets, macrophages, and unidentified extra-hematopoietic cells as possible sources of plasma FXIII-A.^8–11^ Our previous studies suggested that the platelet is not the source of plasma FXIII-A because in 2 severely thrombocytopenic mouse lines, BCl_x_^Plt20/Plt20^ and Mpl^−/−^, plasma FXIII-A levels were normal.^3^ However, a possibility that we did not fully address is that thrombocytopenia per se might induce upregulation of platelet proteins, including FXIII-A, either within the megakaryocyte lineage or within a rescuing cell type. Therefore, in the current study, we have measured FXIII-A in platelets from the Mpl^−/−^ mouse and have investigated the source of plasma FXIII-A by crossing a novel FXIII-A floxed mouse with mice that individually express platelet factor (Pf)4-cre, integrin αM (CD11b)-cre, or lysozyme 2 (LysM)-cre. CD11b is highly expressed on monocytes, inflammatory macrophages, and osteoclasts and is low or absent in certain populations of yolk sac (YS)–derived macrophages,^12,13^ while LysM is expressed on numerous macrophage subpopulations.^14^ The Pf4-cre was designed to delete in the platelet lineage,^15^ but a recent reporter mouse study has also shown Pf4-cre expression within resident macrophages.^16^

In this study, we relate the extent of plasma FXIII-A depletion to the loss of FXIII-A mRNA levels in our cre/lox crosses and deduce that a macrophage population maintains plasma FXIII-A.

## Materials and Methods

Materials and Methods are available in the online-only Data Supplement.

## Results

### Plasma FXIII-A Levels Are Consistent With Either a Platelet or a Myeloid Origin in *cre/lox* FXIII-A-Deficient Mice

**Figure 1.**
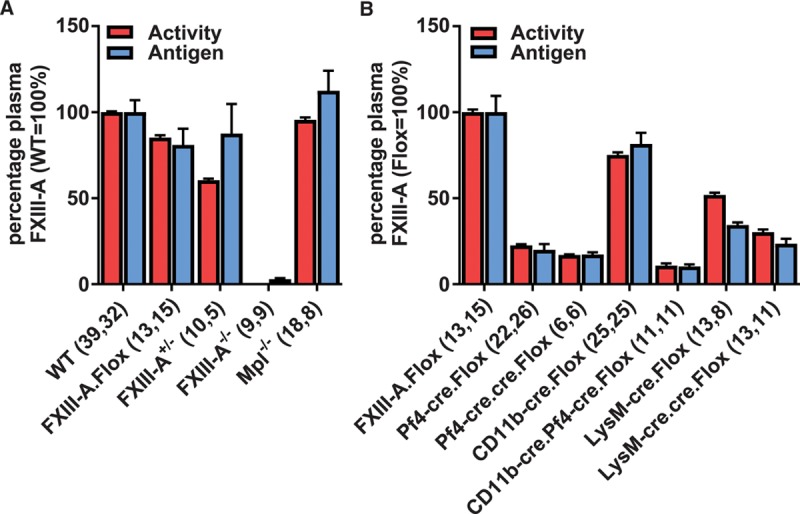
Plasma factor (F)XIII-A activity was measured by biotin-pentylamine incorporation (red bars). Activity measurements were confirmed by immunoblotting FXIII-A protein normalized to α1-antitrypsin (blue bars). **A**, Relates FXIII-A activity in wild-type (WT) mice (100%) to mice in which FXIII-A expression originates from the WT allele and also to the FXIII-A.Flox mouse. **B**, Relates FXIII-A activity in FXIII-A. Flox mice (100%) to crosses between the FXIII-A.Flox and –cre expressing mice. Numbers of separate mouse samples are shown in parentheses. Results are displayed as mean±SEM. Pf4-cre.cre and LysM-cre.cre are homozygous for the respective transgenes. CD11b indicates integrin αM; Flox, FXIII-Aflox/flox; LysM, lysozyme 2; and Pf4, platelet factor 4.

The presence of the flippase recombinase target or LoxP sites within the *F13a1* gene of the floxed mouse (Figure I in the online-only Data Supplement) slightly decreased plasma FXIII-A activity to 85±5% of C57BL/6 wild-type (WT) mice (Figure 1A). Consequently, results are presented either relative to WT mice (Figure 1A) or, where more appropriate, relative to FXIII-A. Flox mice (Figure 1B). Plasma FXIII-A activity was absent in the novel FXIII-A^−/−^ mouse, while plasma FXIII-A activity in heterozygous mice was 61±3% (*P*<0.001; Figure 1A), in agreement with previously described FXIII-A^+/−^ mice.^17^ FXIII-A activity measurements were corroborated by immunoblotting, with generally good agreement between methods (Figure 1).

FXIII-A.Flox mice were crossed with mice expressing *cre* recombinase in megakaryocytes and certain macrophage populations (Pf4-cre^15,16^) or exclusively in myeloid cells (LysM-cre^14^ and CD11b-cre^13^). In Pf4-cre.Flox mice, plasma FXIII-A activity was reduced to 23±3% (*P*<0.001; Figure 1B). To determine whether the residual 23% was explained by suboptimal Pf4-cre expression, we bred mice harboring 2 copies of this transgene, using quantitative polymerase chain reaction of genomic (g)DNA to assess copy number (1 copy normalized to 1.00±0.038, n=12 and 2 copy to 1.83±0.060, n=6).

Pf4-cre.cre.Flox mice did not show a statistically significant further decrease in plasma FXIII-A activity (17±1%, *P*=0.48; Figure 1B), suggesting that incomplete depletion is not explained by suboptimal expression of Pf4-cre.

In CD11b-cre.Flox and LysM-cre.Flox mice, plasma FXIII-A was reduced to 75±5% (*P*=0.003; Figure 1B) and 57±5% (*P*<0.001; Figure 1B), respectively. The LysM-cre knock-in undergoes silencing,^18^ and possibly on account of this, we observed a significant further reduction in plasma FXIII-A (to 30±7%) in 2-copy LysM-cre.cre.Flox mice relative to LysM-cre. Flox mice (*P*<0.001; Figure 1B).

The sum of the plasma FXIII-A reductions achieved individually with Pf4-cre.Flox (77%) and either CD11b-cre.Flox (25%) or LysM-cre.cre.Flox (70%), respectively, equals or exceeds 100%. In contrast, while dual-expressing CD11b-cre.Pf4-cre.Flox mice showed a further reduction in plasma FXIII-A activity (11±2%; Figure 1B) over the Pf4-cre. Flox mice (*P*<0.001), plasma FXIII-A was not completely eliminated. These results suggest that the plasma FXIII-A-releasing cell expresses both Pf4 and myeloid markers.

### Platelet FXIII-A Concentrations in *cre/lox* FXIII-A-Deficient and Thrombocytopenic Mpl^−/−^ Mice Discount the Platelet and Support a Myeloid Origin for Plasma FXIII-A

**Figure 2.**
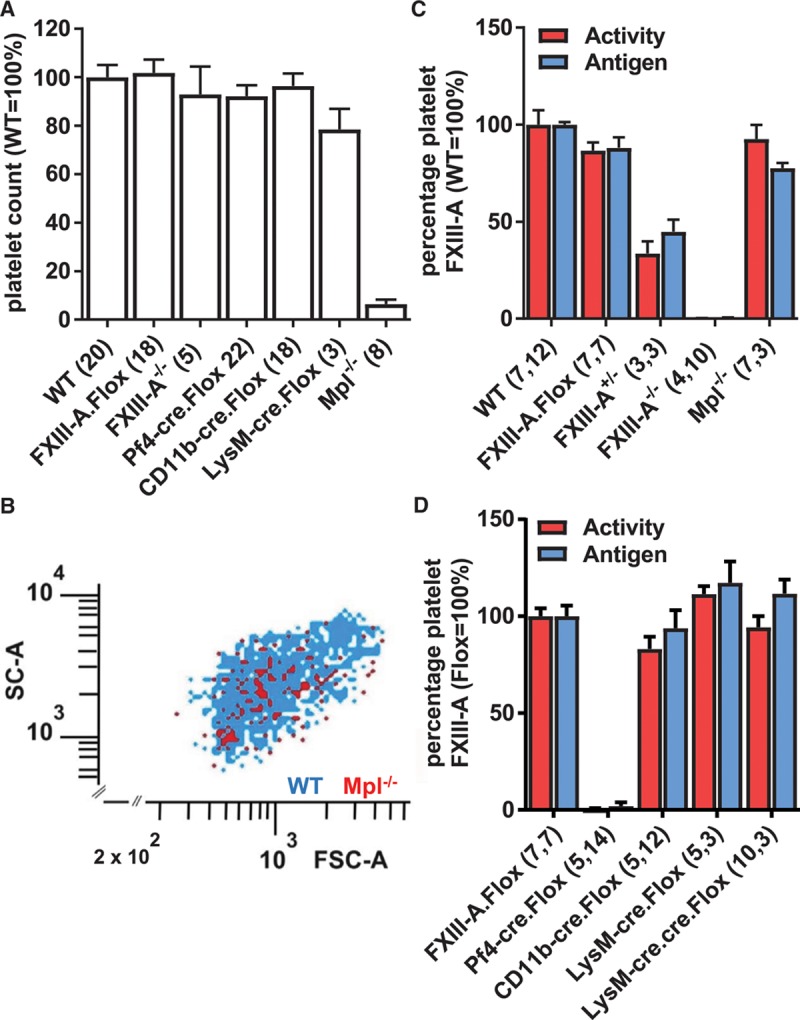
**A**, Platelet counts in whole blood were determined by flow cytometry. **B**, Platelets from wild-type (WT; blue dots) and Mpl^−/−^ mice (red dots) were stained with fluorescein isothiocyanate (FITC)–labeled and phycoerythrin (PE)-labeled anti-GPIbα antibodies, respectively. The plot of forward scatter (FSC-A) vs side scatter (SC-A) shows a similar size distribution in WT and Mpl^−/−^ platelets and is representative of 3 independent experiments. **C** and **D**, Platelet factor (F)XIII-A activity was measured over the linear range by biotin-pentylamine incorporation and adjusted for lactate dehydrogenase (LDH) activity (red bars). Activity measurements were confirmed by immunoblotting, normalized to β-actin (blue bars). Numbers of separate mouse samples are shown in parentheses. Results are displayed as mean±SEM relative to WT (**C**) or FXIII-A.Flox (**D**) as appropriate. CD11b indicates integrin αM; Flox, FXIII-Aflox/flox; LysM, lysozyme 2; and Pf4, platelet factor 4.

Although our previous studies suggested that the platelet was not the source of plasma FXIII-A,^3^ the substantial deletion of plasma FXIII-A in Pf4-cre.Flox mice might be interpreted as indicating that the megakaryocyte–platelet lineage makes a significant contribution to plasma FXIII-A. To further exclude the platelet lineage as the major source of plasma FXIII-A, we demonstrated that Mpl^−/−^ mice have normal plasma FXIII-A activity (95±4%, *P*=0.49; Figure 1A), despite platelet counts in whole blood being 6.4±5% of WT (*P*<0.0001). In contrast, platelet counts did not differ between WT mice and the various cre/lox mice (Figure 2A). We established that the size distribution of Mpl^−/−^ platelets was equivalent to that of WT (Figure 2B) and that FXIII-A activity and protein per platelet were normal (92±6%; Figure 2C). In addition, because thrombocytopenia has been suggested to induce hepatic FXIII-A expression,^9^ we determined by reverse transcriptase polymerase chain reaction that hepatic FXIII-A mRNA was undetectable in either the Mpl^−/−^ or WT mice (C_t_>40). Together, these results both exclude compensatory FXIII-A upregulation in platelets or hepatic cells as a mechanism whereby plasma FXIII-A is maintained and further discount the platelet as the source of plasma FXIII-A.

In the platelet, FXIII-A activity was abolished in Pf4-cre. Flox mice (Figure 2D), showing that single-copy Pf4-cre mice underwent efficient deletion. Moreover, platelet FXIII-A was unaffected in the CD11b-cre.Flox (83±10%), LysM-cre.Flox (111±3%), and LysM-cre.cre.Flox (94±10%) mice (Figure 2D), confirming that these myeloid-cre mice exert their effects on plasma FXIII-A through the myeloid rather than the megakaryocyte lineage. Together, these results suggest that Pf4-cre depletes plasma FXIII-A by acting within myeloid cells, and that these cells are the major site from which plasma FXIII-A is released.

### FXIII-A^pos^ Cardiac Cells Are Pf4-Expressing Macrophages

**Figure 3.**
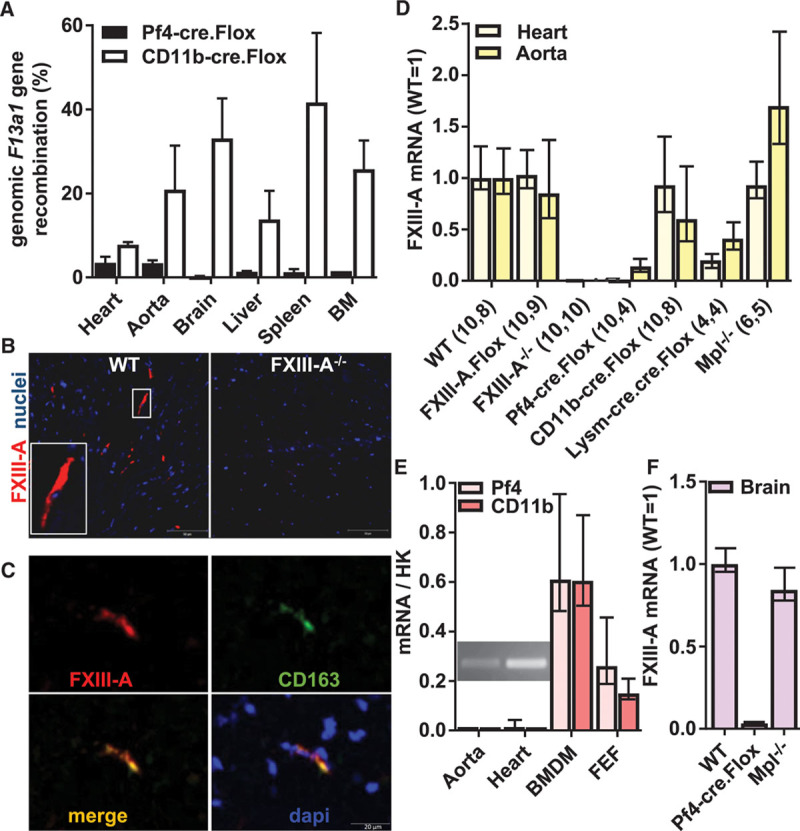
**A**, Genomic *F13a1* recombination in the tissues indicated was determined in Pf4-cre.Flox (dark bars) and CD11b-cre.Flox (white bars) mice. Results are expressed as mean±SEM (n≥3). **B** and **C**, Immunofluorescent staining detects factor (F)XIII-A (red) cells in wild-type (WT) but not FXIII-A^−/−^ heart sections. Nuclei are shown in blue. **B**, A proportion of FXIII-A-labeled cells adopt a spindle-shaped morphology characteristic of cardiac macrophages (inset). Scale bar represents 50 μm. **C**, Some of these FXIII-A^pos^ cells costain for CD163 (green). Scale bar is 5 μm. **D**–**F**, mRNA was isolated from indicated tissues and cDNA subjected to quantitative polymerase chain reaction (qPCR) using housekeeping (HK) transcripts ribosomal protein L32 and β-actin. All results are displayed as mean with 95% confidence interval (CI). **D**, FXIII-A mRNA expression was measured in heart (light yellow bars) and aorta (dark yellow bars) in mice of the genotypes indicated. Numbers of separate mouse samples are shown in parentheses. **E**, Endogenous Pf4 transcripts (light pink bars) in WT aorta and heart (n=4) were measured by reverse transcriptase (RT)-PCR, and reaction products were resolved on agarose gels (inset). Transcript levels were enriched in WT-cultured BM-derived macrophages (BMDM, n=4) at levels similar to endogenous CD11b transcripts (dark pink bars), as well as in a FXIII-A-enriched cardiac cell fraction (FEF, n=4). **F**, FXIII-A mRNA expression was measured in brains (n=3) of young mice of the genotypes indicated. CD11b indicates integrin αM; Flox, FXIII-Aflox/flox; LysM, lysozyme 2; and Pf4, platelet factor 4.

We assayed the extent of *F13a1* genomic deletion using quantitative polymerase chain reaction of gDNA, with reference to a standard curve (Figure II in the online-only Data Supplement). Genomic *F13a1* recombination was essentially as previously described in the organs of CD11b-cre.Flox mice.^13^ In Pf4-cre.Flox mice, genomic deletion totaled 3.5% in heart and 3.4% in aorta and was lower in other organs examined (Figure 3A), excluding the possibility that Pf4-cre expression was promiscuous in Pf4-cre.Flox mice.

We examined the expression of FXIII-A mRNA in heart and aorta, where functional roles of FXIII-A have been described.^19,20^ Immunofluorescence studies revealed FXIII-A^pos^ cells in WT, but not knockout heart (Figure 3B). Some of these FXIII-A^pos^ cells appeared as spindles (inset Figure 3B), in agreement with their previous identification as macrophages.^21^ Moreover, coimmunofluorescence for CD163 and FXIII-A (Figure 3C) and immunohistochemistry of consecutive WT heart sections (Figure III in the online-only Data Supplement) confirmed that ≈80% of the FXIII-A^pos^ cells are CD163^pos^ macrophages (Table 1).

**Table 1.**
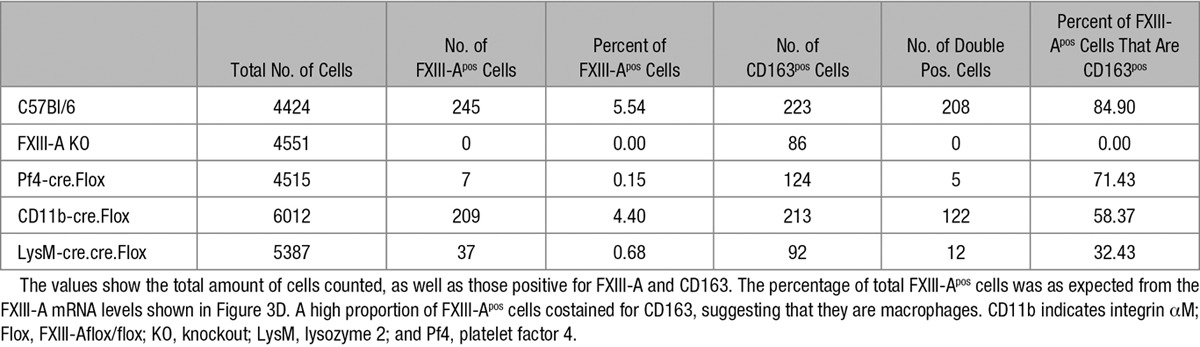
Cardiac Cross Sections From Mice of the Genotypes Indicated Were Immunofluorescently Labeled for CD163 and FXIII-A

FXIII-A mRNA was abolished in heart and depleted in aorta in Pf4cre. Flox mice (Figure 3D). The complete depletion of FXIII-A mRNA in the heart is broadly consistent with the extent of gDNA recombination (3.5%) and the frequency of FXIII-A^pos^ cells (5%; Table 1), provided that Pf4-cre expression is restricted to FXIII-A^pos^ cells. Importantly, in LysM-cre.cre.Flox mice, FXIII-A mRNA was substantially depleted from these tissues, while it was conserved in the Mpl^−/−^ mouse, confirming that FXIII-A^pos^ cells are myeloid rather than platelet/megakaryocyte in origin. As expected, the proportional deletion of the FXIII-A mRNA reflected the loss of FXIII-A^pos^ cells across the various genotypes (Table 1).

Authentic Pf4 transcripts were detected in WT hearts and aorta (insert, Figure 3E), as well as in Mpl^−/−^ hearts (data not shown). Pf4 transcripts were 40-fold enriched in cultured BM-derived macrophages over whole hearts, while the ratio of Pf4 mRNA to CD11b mRNA was comparable between BM-derived macrophages and whole heart (Figure 3E). This is consistent with Pf4 and CD11b mRNA being present at a similar concentration within cardiac macrophages, which represent a minor proportion of the tissue.

Finally, we analyzed cell fractions from collagenase-digested hearts obtained with the Miltenyi neonatal cardiac fibroblast isolation kit. In a fraction that was ≈8-fold enriched in FXIII-A mRNA (FEF) and which represented ≈3% of total heart cell mass, Pf4 and CD11b mRNA were, respectively, 20-fold and 14-fold enriched (Figure 3E). Together, these data indicate that the FXIII-A^pos^ cardiac cells are Pf4-expressing macrophages.

### Plasma FXIII-A Originates From Cells Resembling Aortic Macrophages

In the brain, where because of the blood–brain barrier, macrophages derive exclusively from primitive YS hematopoiesis,^22,23^ we observed that FXIII-A^pos^ cells are present at similar levels in WT and Mpl^−/−^ mice (Figure 3F). This shows that, as in heart and aorta, the FXIII-A^pos^ cells in the brain do not arise from the platelet lineage. Moreover, FXIII-A mRNA was depleted from the brain of the Pf4-cre.Flox mouse (Figure 3F), suggesting that YS-derived macrophages, as BM-derived macrophages, coexpress Pf4 and FXIII-A.

Table 2 shows that the Pf4-cre-mediated reduction in cardiac FXIII-A mRNA (to <1%) greatly exceeds the depletion of the plasma FXIII-A (to ≈20%), making it unlikely that the majority of cardiac cells contribute to the plasma FXIII-A pool. However, in aorta, we observed a similar profile of depletion of FXIII-A mRNA (to ≈14%) to plasma FXIII-A. We, therefore, conclude that plasma FXIII-A is released from cells, similar or identical to those present in the aorta, and that these cells resemble YS-derived macrophages.

**Table 2.**
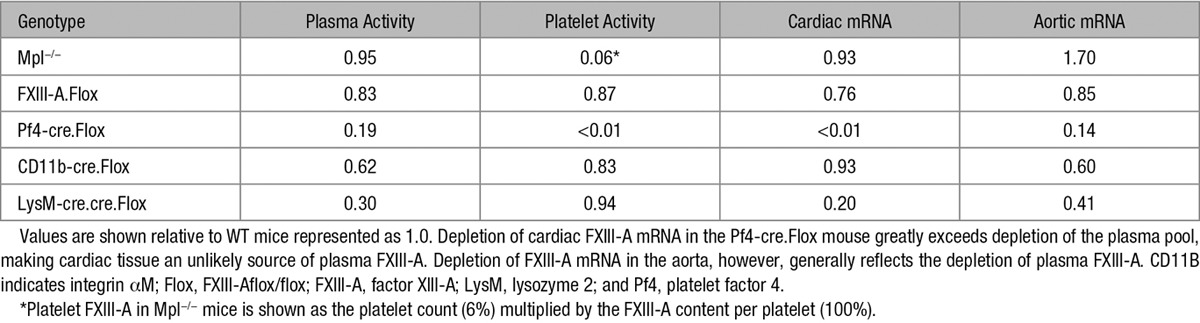
Comparison of Plasma and Platelet FXIII-A Enzyme Activities With FXIII-A mRNA Levels in Heart and Aorta

### BMT Repopulates Resident Macrophages in Heart and Aorta and Reconstitutes Plasma FXIII-A in FXIII-A^−/−^ Mice

**Figure 4.**
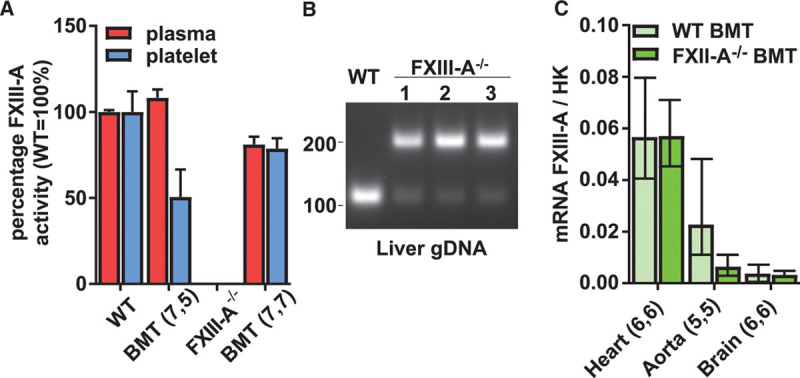
Factor (F)XIII-A^pos^ bone marrow (BM) was transplanted into wild-type (WT) (WT BMT) and FXIII-A^−/−^ (FXIII-A^−/−^ BMT) recipients. **A**, Plasma (red bars) and platelet (blue bars) FXIII-A activity was measured by biotin-pentylamine incorporation assay. Average activities in nontransplanted mice are shown for comparison. Results are presented as mean±SEM. **B**, Livers were excised from a single WT (lane +) and 3 representative FXIII-A^−/−^ (lanes 1, 2, and 3) recipients of WT BM. Amplification of gDNA by polymerase chain reaction (PCR) revealed a preponderance of the deleted allele (200 kDa) in the livers of the FXIII-A^−/−^ recipients and partial repopulation with FXIII-A^+/+^ cells (130 kDa). **C**, FXIII-A mRNA was measured in the hearts, aortas, and brains of WT (light green bars) and FXIII-A^−/−^ (dark green bars) BMT recipients. Results are represented as mean with 95% confidence interval (CI). BMT indicates bone marrow transplant.

BM was transferred from WT mice into irradiated FXIII-A^−/−^ and WT recipients; survival was 7 of 8 in both groups. At 10 weeks post-BMT, FXIII-A^−/−^ recipients expressed normal levels of plasma and platelet FXIII-A (Figure 4A). Recipient genotype at harvest was confirmed by showing that liver gDNA remained predominantly FXIII-A^−/−^, while a minor proportion became FXIII-A^+/+^, consistent with engraftment of donor macrophages (Figure 4B). FXIII-A mRNA expression was detectable in hearts, aortas, and brains of the recipient FXIII-A^−/−^ mice (Figure 4C), the expression in brain being the consequence of irradiation breaching the blood–brain barrier.^22,23^ There was no increase in plasma FXIII-A or FXIII-A mRNA after BMT into WT recipients, suggesting that there are a limited number of niches that become occupied by FXIII-A-expressing cells.

## Discussion

Previously, we demonstrated that plasma FXIII-A levels are normal in the thrombocytopenic Mpl^−/−^ and BCl_x_^Plt20/Plt20^ mice.^3^ However, we did not assay platelet FXIII-A in the Mpl^−/−^ mouse, in which depletion of ≈94% platelets and their precursors results from knockout of the thrombopoietin receptor.^24^ Here, we have shown that the amount of FXIII-A per platelet has not increased to compensate for thrombocytopenia. Platelets arise from fragmentation of megakaryocytes^25^; therefore, cytosolic FXIII-A concentrations in megakaryocytes from the Mpl^−/−^ mouse should also be unchanged. This makes it improbable for platelets and megakaryocytes to be major contributors to the plasma FXIII-A pool, although we cannot exclude a minor contribution.

We next compared the effects of recombination by lineage-specific cre mice on plasma FXIII-A and cellular FXIII-A mRNA levels. Plasma FXIII-A, but not platelet FXIII-A, was partially depleted in CD11b-cre.Flox and LysM-cre.cre.Flox mice, suggesting that macrophages maintain plasma FXIII-A and further excluding the megakaryocyte lineage as a major contributor.

In Pf4-cre.Flox mice, platelet FXIII-A was abolished, while plasma FXIII-A was reduced to 23%. This result further supports a macrophage origin for FXIII-A because a stop/flox study has established that Pf4-cre expression occurs within resident tissue macrophages in addition to megakaryocytes.^16^ Notably, Pf4-cre-mediated recombination within macrophages is mosaic,^16^ potentially accounting for incomplete depletion of plasma FXIII-A. The FXIII-A-expressing cells in brain, heart, and aorta have been previously identified as macrophages,^19,20^ and consistent with this, we show (1) that in heart, as previously demonstrated in skin,^26^ FXIII-A partially co-localizes with the M2 macrophage marker CD163 and (2) that in heart, brain, and aorta, Pf4-cre depleted FXIII-A mRNA.

The expression of Pf4-cre within resident macrophages is likely to be faithful because expression of Pf4 protein was previously demonstrated in adherent cultures of human monocytes^27,28^ and confirmed by us in mouse BM-derived macrophages. In whole heart, as well as in a FXIII-A-enriched cardiac cell fraction, we demonstrated that the ratio of Pf4 to CD11b transcripts was similar to that in BM-derived macrophages, as expected if the Pf4^pos^ cells in the heart are macrophages.

The profile of depletion of plasma FXIII-A in the various cre/lox crosses (Table 2) closely resembles that of aortic FXIII-A mRNA, implicating cells identical or related to aortic resident macrophages as the source of plasma FXIII-A. FXIII-A within aortic macrophages has been implicated in arterial repair and remodeling.^19^ If local release contributes to this function, this could also suggest that aortic macrophages, or cells similar to them, release FXIII-A into the plasma. Similarly, placental macrophages express FXIII-A and may stabilize this organ during pregnancy.^4^ Because intravenous FXIII-A prevents spontaneous abortion,^29^ this shows that the requirement is extracellular and suggests that placental macrophages can release FXIII-A.

The hypothesis that resident macrophages release plasma FXIII-A is consistent with the outcomes of most human BMT protocols.^9–11^ In contrast to Wölpl et al,^8^ Poon et al^11^ observed that conversion from donor to recipient plasma FXIII-A variant occurred over many months after BMT. Further, Inbal et al^9^ and Pihusch et al^10^ observed that BMT caused a much greater decrease in platelet count than plasma FXIII-A, which led Pihusch et al^10^ to suggest that resident macrophages maintain the plasma pool. Notably, alveolar macrophages persist for ≤80 days after BMT.^30,31^ Further, the results of these studies seem inconsistent with release occurring from circulating monocytes, because, like platelets, these cells are short-lived.^32^

BMT of FXIII-A^pos^ cells into FXIII-A^−/−^ mice rescued plasma FXIII-A to normal levels, while transfer into FXIII-A^+/+^ mice caused no further increase in plasma FXIII-A. This may imply that the transplanted cells differentiate within a limited number of niches to become FXIII-A-releasing cells.^33,34^

**Figure 5.**
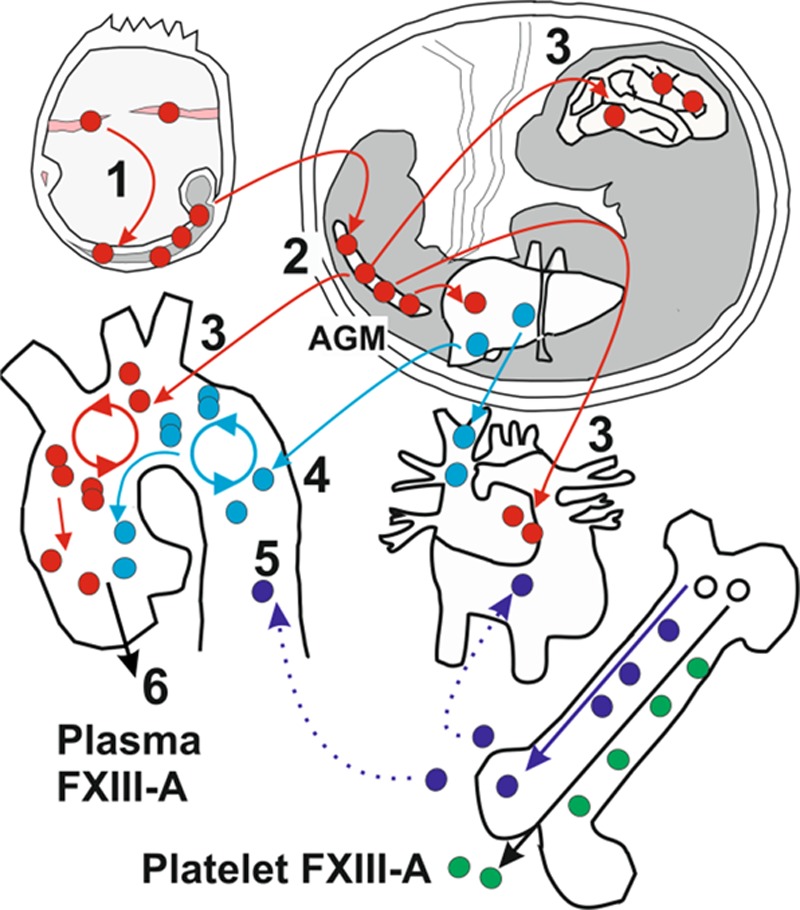
Yolk sac (YS)–derived cells (red) from blood islands (pink) colonize the early embryo (gray; 1) and establish in the aorto-gonad-mesonephros (AGM; 2). Macrophages migrate from the AGM to colonize fetal tissues, including the heart and brain, while some establish in the developing aorta (3). Primitive macrophages persist postnatally. The onset of hematopoiesis within the fetal liver generates monocytes (light blue) that supplement and partially displace YS-derived macrophages (4). Both populations of resident macrophages can maintain themselves within tissues. The onset of hematopoiesis within the BM (white) generates monocytes (dark blue), which supplement the aortic and heart macrophage population over a short time interval (dotted lines). These cells may also differentiate into resident macrophages (5). Plasma factor (F)XIII-A is released from cells similar or identical to aortic resident macrophages (6), and its origin is distinct from platelet FXIII-A (green).

Recent studies have shown that arterial macrophages arise variously from (1) YS macrophages, (2) fetal liver monocytes, and (3) a short wave of BM monocytes.^35^ To determine whether, under normal conditions, monocytes repopulate heart and artery and become FXIII-A-releasing cells, we generated Fms-like tyrosine kinase 3-cre.Flox mice. These recombine in definitive but not primitive hematopoietic cells,^36^ that is, BM and some fetal liver cells^37^ but not YS-derived cells. The Fms-like tyrosine kinase 3-cre transgene becomes fully active only in a minority of mice.^33,38^ One out of 20 Fms-like tyrosine kinase 3-cre.Flox mice underwent efficient recombination and reduced plasma FXIII-A to 34% (Figure IV in the online-only Data Supplement). Assuming that this result is representative, it would imply that a proportion of FXIII-A-releasing cells derive from fetal liver or BM-derived cells, presumably monocytes. This would resemble the situation described for arterial macrophages.^35^ Our model is shown in Figure 5: the aorto-gonad-mesonephros is an early site of hematopoiesis,^39^ and because it is known that vascular macrophages in the early embryo express FXIII-A,^40^ it seems probable that they establish here, persist within the developing aorta, and survive postnatally. Monocyte-derived macrophages subsequently supplement, or partially displace, YS-derived macrophages and contribute to plasma FXIIII-A. Although we have shown that aortic macrophages resemble the plasma FXIII-A-releasing cells, resident macrophages in other tissues may contribute.

The main limitation of the present study is that we have not directly demonstrated that macrophages release FXIII-A. Previously, we detected nonclassical secretion in vitro of interleukin-1β, but not FXIII-A, from IL-4-treated adherent THP-1 cells, which resemble resident macrophages.^41^ In addition, we have detected externalization of the closely related protein transglutaminase 2,^42,43^ but not of FXIII-A, to the surface of mouse macrophages and THP-1 cells (Figure V in the online-only Data Supplement). It may transpire that FXIII-A is released primarily (1) within tissue niches or (2) from Li6C^lo^ resident macrophage precursors,^44^ and that these cells were sparse in culture. Further studies may address these issues by (1) adoptive transfer of defined macrophage precursors, (2) enriching cultures for particular cell types, or (3) establishing coculture models that mimic niches. In conclusion, our studies exclude the platelet and all its precursors as the major source of plasma FXIII-A and instead implicate that resident macrophages maintain plasma FXIII-A.

## Acknowledgments

We sincerely thank Dr Adam Mead, Dr Thomas Boehm, and Dr Warren Alexander for essential animal resources. Dr Warren Alexander, in addition, for our helpful discussions, Dr Neil Turner for the kind gift of mouse cardiac cell fractions, and Eleanor Cawthorne for excellent technical support.

## Sources of Funding

This work was supported by the British Heart Foundation via Programme Grant (RG02/29261) as well as a clinical (KJG FS/11/91/29090) and a nonclinical (KRS FS/13/36/30243) PhD studentship.

## Disclosures

None.

## Supplementary Material

## Notes

Highlights

- Plasma factor XIII-A derives from resident macrophages.
- Plasma factor XIII-A releasing macrophages resemble aortic macrophages.
- Bone marrow–derived monocytes/macrophages can differentiate in situ into plasma factor XIII-A releasing cells.

## References

[1] Fear JD, Miloszewski KJ, Losowsky MS (1983). The half life of factor XIII in the management of inherited deficiency.. Thromb Haemost.

[2] Adány R, Bárdos H (2003). Factor XIII subunit A as an intracellular transglutaminase.. Cell Mol Life Sci.

[3] Cordell PA, Kile BT, Standeven KF, Josefsson EC, Pease RJ, Grant PJ (2010). Association of coagulation factor XIII-A with Golgi proteins within monocyte-macrophages: implications for subcellular trafficking and secretion.. Blood.

[4] Muszbek L, Bereczky Z, Bagoly Z, Komáromi I, Katona É (2011). Factor XIII: a coagulation factor with multiple plasmatic and cellular functions.. Physiol Rev.

[5] Töröcsik D, Bárdos H, Nagy L, Adány R (2005). Identification of factor XIII-A as a marker of alternative macrophage activation.. Cell Mol Life Sci.

[6] Cordell PA, Newell LM, Standeven KF, Adamson PJ, Simpson KR, Smith KA, Jackson CL, Grant PJ, Pease RJ (2015). Normal bone deposition occurs in mice deficient in factor XIII-A and transglutaminase 2.. Matrix Biol.

[7] Kaetsu H, Hashiguchi T, Foster D, Ichinose A (1996). Expression and release of the a and b subunits for human coagulation factor XIII in baby hamster kidney (BHK) cells.. J Biochem.

[8] Wölpl A, Lattke H, Board PG, Arnold R, Schmeiser T, Kubanek B, Robin-Winn M, Pichelmayr R, Goldmann SF (1987). Coagulation factor XIII A and B subunits in bone marrow and liver transplantation.. Transplantation.

[9] Inbal A, Muszbek L, Lubetsky A, Katona E, Levi I, Kárpáti L, Nagler A (2004). Platelets but not monocytes contribute to the plasma levels of factor XIII subunit A in patients undergoing autologous peripheral blood stem cell transplantation.. Blood Coagul Fibrinolysis.

[10] Pihusch R, Salat C, Göhring P, Hentrich M, Wegner H, Pihusch M, Hiller E, Kolb HJ, Ostermann H (2002). Factor XIII activity levels in patients with allogeneic haematopoietic stem cell transplantation and acute graft-versus-host disease of the gut.. Br J Haematol.

[11] Poon MC, Russell JA, Low S, Sinclair GD, Jones AR, Blahey W, Ruether BA, Hoar DI (1989). Hemopoietic origin of factor XIII A subunits in platelets, monocytes, and plasma. Evidence from bone marrow transplantation studies.. J Clin Invest.

[12] Schulz C, Gomez Perdiguero E, Chorro L, Szabo-Rogers H, Cagnard N, Kierdorf K, Prinz M, Wu B, Jacobsen SE, Pollard JW, Frampton J, Liu KJ, Geissmann F (2012). A lineage of myeloid cells independent of Myb and hematopoietic stem cells.. Science.

[13] Ferron M, Vacher J (2005). Targeted expression of Cre recombinase in macrophages and osteoclasts in transgenic mice.. Genesis.

[14] Clausen BE, Burkhardt C, Reith W, Renkawitz R, Förster I (1999). Conditional gene targeting in macrophages and granulocytes using LysMcre mice.. Transgenic Res.

[15] Tiedt R, Schomber T, Hao-Shen H, Skoda RC (2007). Pf4-Cre transgenic mice allow the generation of lineage-restricted gene knockouts for studying megakaryocyte and platelet function in vivo.. Blood.

[16] Pertuy F, Aguilar A, Strassel C, Eckly A, Freund JN, Duluc I, Gachet C, Lanza F, Léon C (2015). Broader expression of the mouse platelet factor 4-cre transgene beyond the megakaryocyte lineage.. J Thromb Haemost.

[17] Lauer P, Metzner HJ, Zettlmeissl G, Li M, Smith AG, Lathe R, Dickneite G (2002). Targeted inactivation of the mouse locus encoding coagulation factor XIII-A: hemostatic abnormalities in mutant mice and characterization of the coagulation deficit.. Thromb Haemost.

[18] Hume DA (2011). Applications of myeloid-specific promoters in transgenic mice support *in vivo* imaging and functional genomics but do not support the concept of distinct macrophage and dendritic cell lineages or roles in immunity.. J Leukoc Biol.

[19] Bakker EN, Pistea A, Spaan JA, Rolf T, de Vries CJ, van Rooijen N, Candi E, VanBavel E (2006). Flow-dependent remodeling of small arteries in mice deficient for tissue-type transglutaminase: possible compensation by macrophage-derived factor XIII.. Circ Res.

[20] Nahrendorf M, Weissleder R, Ertl G (2006). Does FXIII deficiency impair wound healing after myocardial infarction?. PLoS One.

[21] Heidt T, Courties G, Dutta P, Sager HB, Sebas M, Iwamoto Y, Sun Y, Da Silva N, Panizzi P, van der Laan AM, van der Lahn AM, Swirski FK, Weissleder R, Nahrendorf M (2014). Differential contribution of monocytes to heart macrophages in steady-state and after myocardial infarction.. Circ Res.

[22] Samokhvalov IM, Samokhvalova NI, Nishikawa S (2007). Cell tracing shows the contribution of the yolk sac to adult haematopoiesis.. Nature.

[23] Epelman S, Lavine KJ, Randolph GJ (2014). Origin and functions of tissue macrophages.. Immunity.

[24] Alexander WS, Roberts AW, Nicola NA, Li R, Metcalf D (1996). Deficiencies in progenitor cells of multiple hematopoietic lineages and defective megakaryocytopoiesis in mice lacking the thrombopoietic receptor c-Mpl.. Blood.

[25] Machlus KR, Italiano JE (2013). The incredible journey: From megakaryocyte development to platelet formation.. J Cell Biol.

[26] Zaba LC, Fuentes-Duculan J, Steinman RM, Krueger JG, Lowes MA (2007). Normal human dermis contains distinct populations of CD11c+BDCA-1+ dendritic cells and CD163+FXIIIA+ macrophages.. J Clin Invest.

[27] Su AI, Wiltshire T, Batalov S, Lapp H, Ching KA, Block D, Zhang J, Soden R, Hayakawa M, Kreiman G, Cooke MP, Walker JR, Hogenesch JB (2004). A gene atlas of the mouse and human protein-encoding transcriptomes.. Proc Natl Acad Sci U S A.

[28] Schaffner A, Rhyn P, Schoedon G, Schaer DJ (2005). Regulated expression of platelet factor 4 in human monocytes–role of PARs as a quantitatively important monocyte activation pathway.. J Leukoc Biol.

[29] Ivaskevicius V, Seitz R, Kohler HP, Schroeder V, Muszbek L, Ariens RA, Seifried E, Oldenburg J, Study Group (2007). International registry on factor XIII deficiency: a basis formed mostly on European data.. Thromb Haemost.

[30] Thomas ED, Ramberg RE, Sale GE, Sparkes RS, Golde DW (1976). Direct evidence for a bone marrow origin of the alveolar macrophage in man.. Science.

[31] Hashimoto D, Chow A, Noizat C (2013). Tissue-resident macrophages self-maintain locally throughout adult life with minimal contribution from circulating monocytes.. Immunity.

[32] Yona S, Kim KW, Wolf Y, Mildner A, Varol D, Breker M, Strauss-Ayali D, Viukov S, Guilliams M, Misharin A, Hume DA, Perlman H, Malissen B, Zelzer E, Jung S (2013). Fate mapping reveals origins and dynamics of monocytes and tissue macrophages under homeostasis.. Immunity.

[33] Boyer SW, Beaudin AE, Forsberg EC (2012). Mapping differentiation pathways from hematopoietic stem cells using Flk2/Flt3 lineage tracing.. Cell Cycle.

[34] Molawi K, Wolf Y, Kandalla PK (2014). Progressive replacement of embryo-derived cardiac macrophages with age.. J Exp Med.

[35] Ensan S, Li A, Besla R (2016). Self-renewing resident arterial macrophages arise from embryonic CX3CR1(+) precursors and circulating monocytes immediately after birth.. Nat Immunol.

[36] Benz C, Martins VC, Radtke F, Bleul CC (2008). The stream of precursors that colonizes the thymus proceeds selectively through the early T lineage precursor stage of T cell development.. J Exp Med.

[37] Hoeffel G, Chen J, Lavin Y (2015). C-Myb(+) erythro-myeloid progenitor-derived fetal monocytes give rise to adult tissue-resident macrophages.. Immunity.

[38] Beaudin AE, Boyer SW, Forsberg EC (2014). Flk2/Flt3 promotes both myeloid and lymphoid development by expanding non-self-renewing multipotent hematopoietic progenitor cells.. Exp Hematol.

[39] Sugiyama D, Inoue-Yokoo T, Fraser ST, Kulkeaw K, Mizuochi C, Horio Y (2011). Embryonic regulation of the mouse hematopoietic niche.. ScientificWorldJournal.

[40] Kappelmayer J, Bacskó G, Birinyi L, Zákány R, Kelemen E, Adány R (1995). Consecutive appearance of coagulation factor XIII subunit A in macrophages, megakaryocytes, and liver cells during early human development.. Blood.

[41] Varol C, Mildner A, Jung S (2015). Macrophages: development and tissue specialization.. Annu Rev Immunol.

[42] Piacentini M, D’Eletto M, Farrace MG, Rodolfo C, Del Nonno F, Ippolito G, Falasca L (2014). Characterization of distinct sub-cellular location of transglutaminase type II: changes in intracellular distribution in physiological and pathological states.. Cell Tissue Res.

[43] van den Akker J, van Weert A, Afink G, Bakker EN, van der Pol E, Böing AN, Nieuwland R, VanBavel E (2012). Transglutaminase 2 is secreted from smooth muscle cells by transamidation-dependent microparticle formation.. Amino Acids.

[44] Ramachandran P, Pellicoro A, Vernon MA (2012). Differential Ly-6C expression identifies the recruited macrophage phenotype, which orchestrates the regression of murine liver fibrosis.. Proc Natl Acad Sci U S A.

