# Supplementary material for: Cre/lox Studies Identify Resident Macrophages as the Major Source of Circulating Coagulation Factor XIII-A
Source: Arterioscler Thromb Vasc Biol. 2017 Jul 26;37(8):1494–502. doi: 10.1161/ATVBAHA.117.309271 (PMC5526434; doi:10.1161/ATVBAHA.117.309271)
Supplement: Supplementary file 1 [file atv-37-1494-s001.pdf]

Beckers et al Supplemental Materials and Methods

Cre/lox studies identify resident macrophages as the major source of circulating coagulation Factor XIII-A.

Cora M.L. Beckers[1*], Kingsley R. Simpson[1*], Kathryn J. Griffin[1*], Jane M. Brown[1], Lih T. Cheah[1], Kerrie A. Smith[1], Jean Vacher[2], Paul A. Cordell[1], Mark T. Kearney[1], Peter J. Grant[1#] and Richard J. Pease[1#]

[1]Leeds Institute for Cardiovascular and Metabolic Medicine, LIGHT laboratories, University of Leeds, United Kingdom
[2]Clinical Research Institute of Montreal, Department of Medicine, McGill University, Montréal, Québec, Canada

[*]CMLB., KRS. and KJG. These authors contributed equally to this article.
[#]PJG. and RJP. These authors share senior authorship.

Running title: Resident macrophages maintain plasma FXIII-A

Correspondence to Richard Pease, PhD, Leeds Institute of Cardiovascular and Metabolic Medicine, The LIGHT laboratories, University of Leeds, Clarendon Way, Leeds LS29JT, UK. E-mail: R.J.Pease@Leeds.ac.uk Phone: +44 113 343 7740.

Breeding of mouse lines.
Animal housing, husbandry and procedures were conducted in accordance with guidelines and regulations of the University of Leeds and United Kingdom Home Office. Mice had free access to laboratory chow and water. A C57/129 mixed background mouse with LoxP sites inserted into exon 8 (coding exon 7, which includes the catalytic cysteine residue) of a single *F13a1* allele was generated by targeted recombination of embryonic stem cells from mouse strain 129 (GenOway, Lille). Following selection of G418 resistant clones and indication that appropriate recombinant ES cells had been obtained, these cells were used to generate chimeric blastocysts with cells from C57BL/6 mice. Chimeric offspring were then mated with C57BL/6 mice expressing flippase (flp) recombinase to generate progeny in which the neomycin phosphotransferase cassette had been excised from the majority of cells to leave a single flp recognition target (FRT) site (supplementary Figure I). Mice mosaic for the flp deletion were backcrossed with C57BL/6 wild-type mice to generate true FXIII-$A^{+/flox}$ heterozygotes. Liver gDNA from the FXIII-$A^{+/flox}$ mouse was amplified by PCR across the entire FXIII-A floxed locus using primers 5'-TGCTGGTGTCTTTAACACATTTTTAA-3' and 5'-TGGGCCGAGAATGAATTGGT-3'. The product was sub-cloned into vector pCR4-TOPO (Invitrogen) and subjected to dideoxy sequencing to verify the expected sequences in the WT and floxed alleles (DNA Sequencing & Services, College of Life Sciences, University of Dundee, Scotland).
A FXIII-$A^{+/-}$ heterozygous mouse was also derived from the FXIII-$A^{+/flox}$ mouse by crossing with C57BL/6 transgenic mice expressing CMV-cre recombinase to generate partially deleted mice and then crossing these mosaic mice with C57BL/6 wild-type mice to obtain true heterozygotes (GenOway).
Both the FXIII-$A^{+/flox}$ and the FXIII-$A^{+/-}$ mouse lines were backcrossed against C57BL/6 mice to enrich the background to >97.5% C57BL/6 before breeding the floxed or knockout allele to homozygosity. FXIII-$A^{-/-}$ mice were in all cases obtained as the offspring of FXIII-$A^{+/-}$ females mated with FXIII-$A^{-/-}$ males, since as previously reported,[1] female FXIII-$A^{-/-}$ mice did not survive pregnancy.
The LysM-cre mouse[2] (Charles River, UK) is a knock-in of cre recombinase into the macrophage-specific lysozyme 2 gene and so its expression is expected to reflect the endogenous gene. The Pf4-cre mouse[3] (Charles River, UK) is transgenic for a bacterial artificial chromosome construct in which the Pf4 coding sequence has been replaced by cre recombinase, with flanking genes present to promote fidelity of expression. The Flt3-cre mouse[4] is transgenic for a bacterial artificial chromosome construct in which the FMS-like tyrosine kinase (Flt3) coding sequence is replaced with cre recombinase and was supplied by Dr Adam Mead (University of Oxford), with kind permission from Dr Thomas Boehm (University of Freiburg). The CD11b-cre mouse has been previously described and is transgenic for a cre construct under the control of a minimal promoter.[5] All cre-recombinase expressing mice were obtained on a C57BL/6 background. $Mpl^{-/-}$ mice[6] were a kind gift of Dr Warren Alexander (Walter and Eliza Hall Institute of Medical Research). The genotypes of all mice listed above were determined by PCR amplification of DNA from ear notches obtained at 4 weeks that were solubilised for 20min at 95°C in 100µl of 25mM NaOH / 0.2mM EDTA prior to neutralisation. Oligonucleotides used for routine mouse genotyping and the sizes of products obtained are shown in supplementary Table I.
In some cases, mice were bred with two copies of Pf4-cre and needed to be distinguished from mice harbouring a single copy. Since the integration site of the transgene has not been reported, the copy number of the Pf4-cre construct was obtained by designing 3 primers, one in the Pf4 promoter sequence common to the authentic Pf4 gene and the transgene, and one each specific to the authentic gene or the transgene. This enabled qPCR of the transgene relative to the endogenous Pf4 gene using 60ng of total gDNA (Supplementary Table I), 10µL of the Roche LightCycler 480 SYBR Green I Master Mix and 500nM of each primer in a total volume of 20µL. Reaction conditions used (95°C for 10min, 45 cycles of 95°C for 20s, 64°C for 20s and 72°C for 45s) were followed by a melting curve from 65°C to 95°C, giving a ΔC_t which was converted into an amount of gene product by the Livak $2^{-\Delta Ct}$ method.[7]

Blood sampling and organ harvest.
Blood samples from anaesthetised mice (8-12 weeks) were drawn directly into 250µl citrate, theophylline, adenosine, and dipyridamole (CTAD) anticoagulant (Becton Dickinson). Phosphate buffered saline (PBS, Gibco) perfused organs were harvested into liquid nitrogen prior to DNA/RNA analysis.

Bone Marrow Transplantation.
Female C57BL/6 (WT) and FXIII-A$^{-/-}$ mice at 7 weeks of age and were placed on water containing enrofloxacin (50µg/ml, Bayer) and subjected to total body irradiation (8.45 Gy) at 8 weeks. BM cells from WT male donors (1.0x10^{6}) were transplanted 24h after irradiation. Blood and organs were harvested 10 weeks later.

Platelet counting.
Blood/CTAD mixture was diluted 20-fold in resuspension buffer (50mM Tris, 100mM NaCl, pH7.4) containing 0.1U/ml apyrase (Sigma-Aldrich) and 1µg/ml prostaglandin E-1 (Calbiochem). Platelets stained with 20% vol/vol fluorescein isothiocyanate (FITC)-labelled rat anti-mouse glycoprotein (GP)Ibα (Emfret Analytics, Germany) were counted on a Becton Dickinson Aria II cell sorter. Platelet size in WT and Mpl$^{-/-}$ mice was compared by separate labelling with GPIbα-FITC or GPIbα-phycoerythrin (PE).

Platelet harvest.
Platelet rich plasma from ~1.0ml of blood/CTAD was obtained by centrifugation for 5min at 200xg. Platelet pellets, obtained from platelet rich plasma by centrifugation at 1000xg for 10min, were dispersed in resuspension buffer. Platelet pellets, were harvested by cell sorting (5x10^{6} /aliquot) in initial experiments and in later experiments by centrifugation alone, were washed and stored at -80˚C. Platelet depleted plasma was stored at -40˚C.

FXIII-A activity assay.
Plasma FXIII-A activity was measured by a modified biotin-pentylamine incorporation assay[8]. To ensure that each individual sample was assayed in the linear range of the assay, it was measured at 3 concentrations and in triplicate at each concentration. The middle value was used for analysis, provided the upper and lower samples were in proportion. FXIII-A activity of detergent-lysed platelets was assayed in duplicate reactions containing 10^{5}, 10^{6} and 2.5x10^{6} cells. In the first instance, flow-sorted platelets were used but subsequently it was confirmed that washed platelets could substitute. A reference sample of human plasma plus a plasma sample from a WT mouse were included throughout. Lactate dehydrogenase was assayed (Cytotox96 kit, Promega) to adjust for variation in platelet yield. The increased volume of the flow sorted platelet samples (100µl for assay) necessitated adjustment of reagent volumes. Plates (96 well, Nunc Maxisorb) were coated overnight with 100µl per well (plasma assay) or 200µl (platelet assay) of fibrinogen (40µg/ml, Calbiochem) in TBS (40mM Tris, 40mM NaCl, pH8.3) and blocked with 1% bovine serum albumin in TBS for 90min at 37°C. Samples were incubated with plasma (150µl) or platelet (100µl) reaction mix for 7.5 min at 25°C; (Plasma/platelet mix: TBS/TBS plus 0.2% Tween-20 containing 110µM/220µM dithiothreitol (Sigma Aldrich), 333µM/1332µM biotin-pentylamine (Pierce), 11mM/22mM $CaCl_2$, 1.1U/2.2U.ml^{-1} thrombin (Calbiochem)). Reactions were quenched with 200µl of 200mM ethylenediamine tetra-acetic acid (EDTA), incubated for 1h at 37°C with 100µl (plasma assay) or 200µl (platelet assay) of alkaline phosphatase-labelled streptavidin (2µg/ml, Sigma Aldrich) in TBS containing 1% bovine serum albumin and 0.1% Tween-20. Subsequently, 100µl of 2.7mM 4-nitrophenol phosphate (Sigma) in 1M diethanolamine pH9.8 was added and A_{405nm} was recorded every min over the linear range at 30˚C. Mice of either genders were used, except for Flt3-cre[4] and CD11b-cre[5] mice, which carry the transgene on the Y-chromosome.

FXIII-A antigen assay.
Plasma samples and platelet pellets (10x10^6 cells) were mixed 1:4 with NuPAGE loading buffer (Life Technologies), resolved on 8% SDS gels and transferred to nitrocellulose membranes (Bio-Rad).[9] Membranes were probed with sheep anti-FXIII-A antibody SAF13A (Enzyme Research Laboratories), mouse anti-β-actin (Sigma Aldrich) or chicken anti-α1 antitrypsin (Immune systems), washed and incubated with rabbit anti-sheep-, rabbit anti-mouse- (Dako) and goat anti-chicken (Abcam) horseradish peroxidase, respectively. Immunoreactive bands were detected by chemiluminescence (Clarity, Bio-Rad) and analysed using the Fuji Science Imaging System with AIDA Image Analyzer software (Raytest Isotopenmessgeräte). For each assay the FXIII-A/loading control ratios of the cre/lox crosses were compared to the FXIII-A/loading control ratio of a WT sample which was present on the same gel.

Macrophage culture.
Epiphyses were removed from bones rinsed in 70% ethanol and PBS and BM cells harvested in RPMI medium containing 100U/ml penicillin, 0.1mg/ml streptomycin and 0.25µg/ml amphotericin B. Homogenised extracts were passed through a 70µm filter, pelleted and resuspended in RPMI containing 20% foetal calf serum, 2nmol/ml L-glutamine and 50ng/ml macrophage colony stimulating factor-1. Cells were plated at a density of 4x10^6.ml^{-1}. At day 4, 10ml complete RPMI was added. Washed cells were snap frozen at day 7, in preparation for RNA analysis.

Quantitative PCR.
Nucleic acids were released into TRIzol (ThermoFisher) using a TissueLyser II (Qiagen) for organ samples or by gentle mixing to disperse cultured cells. Nucleic acids were precipitated with 100% ethanol and contaminating DNA removed with the DNA-free kit (ThermoFisher). RNA was transcribed to cDNA using the High Capacity Reverse Transcription Kit (ThermoFisher). qPCR was carried out in duplicate with a commercial SyBr Green PCR mix (Roche) using a Lightcycler 480. Amplification cycles (95°C for 10 min, 45 cycles of 95°C for 10 sec, 60°C for 1 min) were followed by a melting curve to ensure a single product was amplified. β-actin and ribosomal protein L32 (RPL32) were used as housekeeping controls.

Immunofluorescent detection of FXIII-A and CD163 on heart sections.
Mouse hearts were either perfused with 4% paraformaldehyde in PBS, excised and processed for paraffin embedding or were frozen in OCT compound and stored at -80 degrees prior to processing. FXIII-A detection was performed on 4µm cross sections using sheep anti FXIII-A (Enzyme Research Laboratories) followed by donkey anti sheep conjugated with Alexa Fluor 555 (ThermoFisher) while CD163 detection was performed with rabbit anti CD163 (M-96, Santa Cruz) with donkey anti rabbit conjugated with Alexa Fluor 488 (ThermoFisher). Slides were sealed using Vectashield containing DAPI (Vector Laboratories). Fluorescence imaging microscopy was performed using an Olympus BX61WI inverted microscope with an XC10-IR camera under the control of CellSens software (Olympus).

Immunohistochemical detection of FXIII-A and CD163 on consecutive heart sections.
WT mouse hearts and livers and FXIII-A KO hearts were perfused with 4% paraformaldehyde in PBS, excised and processed for paraffin embedding. Consecutive cross sections (4µm) were deparaffinised, rehydrated and stained for either FXIII-A or CD163. FXIII-A KO hearts sections were used as a negative staining control. Heart sections of FXIII-A KO mice were used as negative controls for the FXIII-A staining. WT liver sections were used as the recommended positive control for CD163 staining, while sections without primary antibody were used as negative controls. For FXIII-A detection peroxidase activity was blocked using 30% H_2O_2 in methanol and incubated in rabbit serum from the Vectastain ABC-HRP kit (Vectorlab PK-4006). Sections were incubating with sheep anti FXIII-A (Enzyme Research Laboratories) and an Avidin/HRP biotinylated rabbit anti sheep antibody. Diaminobenzidene (DAB) substrate (Vectorlab SK-4100) was used for visualisation. For CD163 staining antigen

retrieval was performed by pressure cooking for 2 min in 100mM Tris, 1 mM EDTA, pH 9. Sections were blocked in goat serum from the Vectastain ABC-AP kit (Vectorlab AK-5001). An additional Avidin B blocking was performed and sections were incubated with rabbit anti CD163 (M-96) (Santa Cruz 33560) and an Avidin/alkaline phosphatase biotinylated goat anti rabbit antibody. Alkaline phosphatase substrate 3-amino-9-ethylcarbazol (red, Vectorlab SK-5001) was used for visualisation. Slides were counterstained with haematoxylin, dehydrated and mounted. Brightfield microscopy was performed using an Olympus BX61WI inverted microscope with an XC10-IR camera under the control of CellSens software (Olympus). The proportion of FXII-A^{pos} to FXIII-A^{neg} heart cells was counted in 3 representative images.

Immunofluorescent detection of FXIII-A and TG2 in human macrophages.

Peripheral blood mononuclear cells were isolated from human blood using Ficoll Paque (GE Healthcare) and monocytes were subsequently purified using anti CD14 MACS beads (Miltenyi Biotec) following manufacturers' protocols. Cells were cultured for 6 days on glass coverslips in macrophage serum-free medium (Gibco/Thermo) containing 20ng/ml recombinant macrophage colony stimulating factor (M-CSF, eBioscience). THP-1 cells (acquired from ECACC) were cultured in RMPI 1640 medium supplemented with 10% foetal calf serum (PAA labs), glutamine (2mM) and antibiotic/antibiotic mix (Sigma). Prior to analysis, cells were incubated with phorbol myristate acetate (PMA) at 500ng/ml for 3h, washed three times in Dulbecco's modified phosphate-buffered saline (DPBS), seeded onto glass coverslips then cultured for 3 days.

For cell surface labelling, cells were placed on ice, washed 3 times with ice-cold DPBS and blocked for 30 min with DBPS containing 2% w/v bovine serum albumin (BSA) and a 1:10 dilution of Fc Receptor blocker solution (Miltenyi Biotec). Cells were rinsed in DPBS/BSA and incubated for 1h with primary antibodies diluted in DPBS/BSA (anti FXIII-A: SAF13A-AP 2µg/ml (Enzyme Research Laboratories), anti TG2 mixed monoclonal antibodies CUB7402 and TG100 1µg/ml (Thermo Fisher), anti giantin rabbit polyclonal antiserum 1:1000, ab24586 (Abcam)). After 3 further washes in DPBS/BSA, cells were incubated for 45 minutes with 0.5µg/ml multiple labelling grade secondary antibodies (DyLight 488 donkey anti rabbit, DyLight 549 donkey anti sheep and DyLight 649 donkey anti mouse (Jackson Immunoresearch)), washed 3 times with DPBS/BSA and twice with DPBS before fixation with warmed 3% paraformaldehyde in DBPS (pH7.4). Subsequently samples were washed 3 times in DBPS, quenched for 10 minutes in 50mM NH_4Cl in DPBS and washed a further 3 times in DPBS. Coverslips were mounted using ProLong Gold antifade with DAPI (Invitrogen) and single 1µm thick confocal sections through the centre of the cell were imaged on a Zeiss LSM510 inverted laser-scanning confocal microscope using a 63x oil 1.4NA objective at 1024x1024 resolution.

Statistics.

For all assays performed n was ≥3 unless otherwise stated. Data are reported as mean ± SEM unless otherwise indicated. ANOVA tests were followed by Student *t* tests. P values below 0.05 were considered significant.

Reference List

(1) Koseki-Kuno S, Yamakawa M, Dickneite G, Ichinose A. Factor XIII A subunit-deficient mice developed severe uterine bleeding events and subsequent spontaneous miscarriages. *Blood* 2003 December 15;102:4410-2.

(2) Clausen BE, Burkhardt C, Reith W, Renkawitz R, Forster I. Conditional gene targeting in macrophages and granulocytes using LysMcre mice. *Transgenic Res* 1999 August;8:265-77.

(3) Tiedt R, Schomber T, Hao-Shen H, Skoda RC. Pf4-Cre transgenic mice allow the generation of lineage-restricted gene knockouts for studying megakaryocyte and platelet function in vivo. *Blood* 2007 February 15;109:1503-6.

(4) Benz C, Martins VC, Radtke F, Bleul CC. The stream of precursors that colonizes the thymus proceeds selectively through the early T lineage precursor stage of T cell development. *J Exp Med* 2008 May 12;205:1187-99.

(5) Ferron M, Vacher J. Targeted expression of Cre recombinase in macrophages and osteoclasts in transgenic mice. *Genesis* 2005 March;41:138-45.

(6) Alexander WS, Roberts AW, Nicola NA, Li R, Metcalf D. Deficiencies in progenitor cells of multiple hematopoietic lineages and defective megakaryocytopoiesis in mice lacking the thrombopoietic receptor c-Mpl. *Blood* 1996 March 15;87:2162-70.

(7) Livak KJ, Wills QF, Tipping AJ, Datta K, Mittal R, Goldson AJ, Sexton DW, Holmes CC. Methods for qPCR gene expression profiling applied to 1440 lymphoblastoid single cells. *Methods* 2013 January;59:71-9.

(8) Song YC, Sheng D, Taubenfeld SM, Matsueda GR. A microtiter assay for factor XIII using fibrinogen and biotinylcadaverine as substrates. *Anal Biochem* 1994 November 15;223:88-92.

(9) Beckers CM, Garcia-Vallejo JJ, van Hinsbergh VW, van Nieuw Amerongen GP. Nuclear targeting of beta-catenin and p120ctn during thrombin-induced endothelial barrier dysfunction. *Cardiovasc Res* 2008 September 1;79:679-88.
