# Supplementary material for: Cre/lox Studies Identify Resident Macrophages as the Major Source of Circulating Coagulation Factor XIII-A
Source: Arterioscler Thromb Vasc Biol. 2017 Jul 26;37(8):1494–502. doi: 10.1161/ATVBAHA.117.309271 (PMC5526434; doi:10.1161/ATVBAHA.117.309271)
Supplement: Supplementary file 2 [file atv-37-1494-s002.pdf]

Beckers et al Supplemental Figures

Cre/lox studies identify resident macrophages as the major source of circulating coagulation Factor XIII-A.

Cora M.L. Beckers[1*], Kingsley R. Simpson[1*], Kathryn J. Griffin[1*], Jane M. Brown[1], Lih T. Cheah[1], Kerrie A. Smith[1], Jean Vacher[2], Paul A. Cordell[1], Mark T. Kearney[1], Peter J. Grant[1#] and Richard J. Pease[1#]

[1]Leeds Institute for Cardiovascular and Metabolic Medicine, LIGHT laboratories, University of Leeds, United Kingdom
[2]Clinical Research Institute of Montreal, Department of Medicine, McGill University, Montréal, Québec, Canada

[*]CMLB., KRS. and KJG. These authors contributed equally to this article.
[#]PJG. and RJP. These authors share senior authorship.

Running title: Resident macrophages maintain plasma FXIII-A

Correspondence to Richard Pease, PhD, Leeds Institute of Cardiovascular and Metabolic Medicine, The LIGHT laboratories, University of Leeds, Clarendon Way, Leeds LS29JT, UK. E-mail: R.J.Pease@Leeds.ac.uk Phone: +44 113 343 7740.

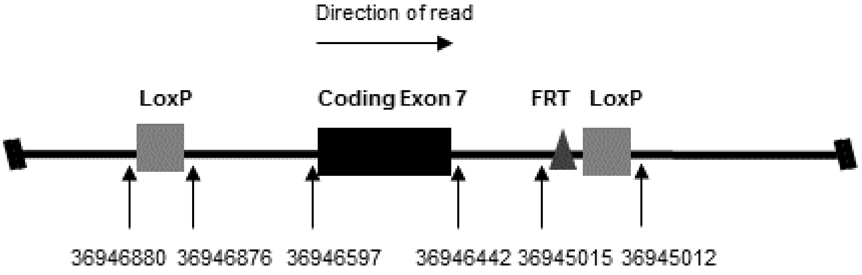

Supplementary Figure I. Lox P sites were inserted into the mouse *F13a1* gene to span coding exon 7 (genomic exon 8), at the sites numbered according to the NCBI reference genome for the C57BL/6 mouse strain, NC000079.6 (chromosome 13). A FRT site remains after flp recombinase-mediated excision of the neomycin phosphotransferase cassette that was used for selection.

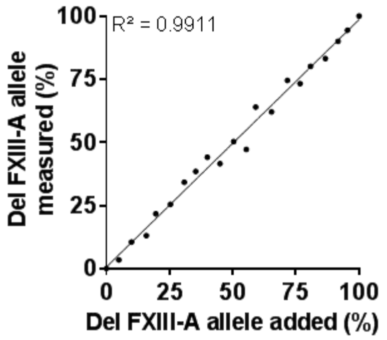

Supplementary Figure II. Genomic DNA, isolated from the livers of FXIII-A$^{Flox/Flox}$ mice and from FXIII-A$^{-/-}$ mice (CMV-cre recombined FXIII-A$^{Flox/Flox}$ mice), was mixed in known proportions and subjected to quantitative PCR to establish a calibration curve, using primer pairs shown in Supplementary Table 1.

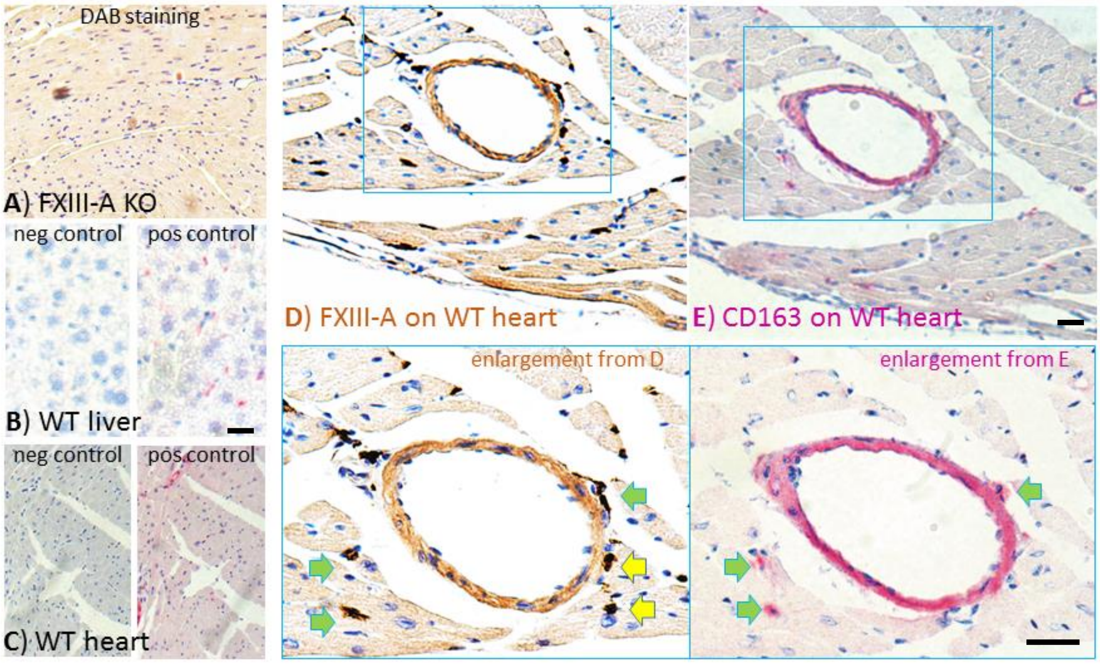

Supplementary Figure III. A) FXIII-A staining on FXIII-A$^{-/-}$ heart section is shown as a negative control (no antigen) for the DAB staining protocol. **B**) Negative (no primary antibody) and positive controls for CD163 alkaline phosphatase staining (red) on liver sections. **C**) Negative (no primary antibody) and positive controls for CD163 staining on heart sections. **D**) FXIII-A and **E**) CD163 staining of consecutive WT heart sections. FXIII-A positive cells (brown) that are also clearly CD163 positive (pink) are indicated in the enlargements of D and E by green arrows. In the case of other FXIII-A positive cells co-staining with CD163 cannot be determined (yellow arrows). This suggests that a proportion of the FXIII-A positive cells in the heart are also CD163 positive. Scale bars represent 50μm.

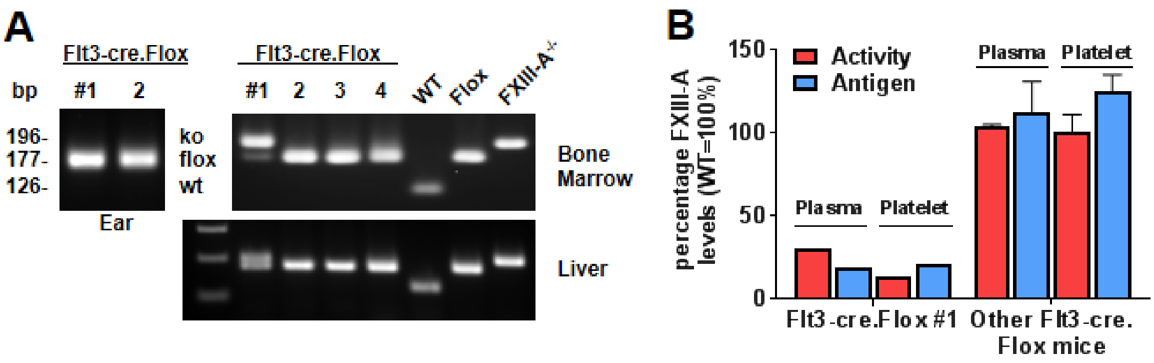

Supplementary Figure IV. A) The gDNA from the ear, liver and bone marrow of Flt3-cre.Flox mice was analysed by PCR, together with gDNA from mice of the genotypes shown. In a single Flt3-cre.Flox mouse (#1, which showed no recombination in ear notch gDNA) efficient recombination of the *F13a1* gene occurred in bone marrow, while in Flt3-cre.Flox mice #2,3,4 minimal or no recombination occurred. In mouse #1, ~50% recombination occurred in liver, in line with reports that a high proportion of adult liver cells are Flt3-dependent.[1] The 196 base pair (bp) band represents the KO allele, the 177bp the floxed allele and the 126bp band the WT allele. **B)** Plasma and platelet FXIII-A activity (red bars) and protein (blue bars) levels were decreased in the efficiently recombined Flt3-cre.Flox mouse #1, but not in the other, non-recombined mice of the same genotype. Results are displayed as mean±SEM.

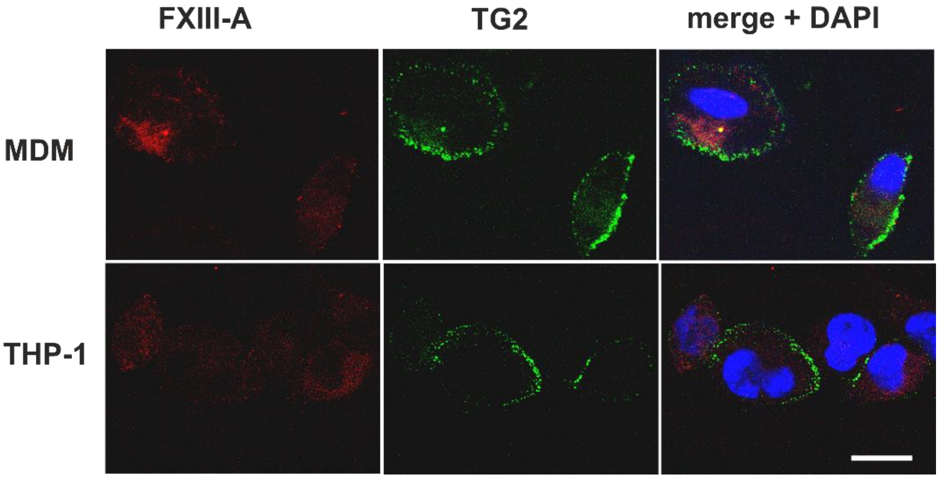

Supplementary Figure V. Cultured adherent human monocyte-derived macrophages (MDM) or human monocytic leukaemic cells (THP-1) were treated with antibodies to TG2 (green) or FXIII-A (red), under conditions where cell integrity was preserved (as confirmed by the absence of giantin staining, not shown). Cell nuclei detected with DAPI are shown in blue. Intact cells showed a ring of TG2 staining on the outer face of the plasma membrane. Very feint staining with the anti-FXIII-A antibody did not exceed the isotype control, and did not reveal cell-surface labelling. As previously observed,[2] cytosolic FXIII-A staining was apparent in permeabilised cells, while permeabilisation also resulted in staining of the Golgi apparatus with giantin (not shown). Using acquisition settings optimised for experimental samples, no labelling of cells was detected when primary antibodies were omitted from the staining procedure. Scale bar represent 20µm.

Supplementary Table I

**Genotyping**	**Primer sequence, 5' to 3'.**	**Target**
FXIII-A fwd	TCTGGGCCAAACCAAGTACCTGG	
FXIII-A rev1	CAAGACCAGACTGTGCAAAGGG	WT allele 126bp, floxed allele 177bp
FXIII-A rev2	GGGGACTTGCTCCCATGTAAA	cre-deleted allele 199bp
PF4-cre fwd	TGGGCAGGCAGTGAAGATAA	
PF4-cre rev1	CATGTCAAGAGGGTGCCACTGGA	endogenous gene 192bp
PF4-cre rev2	ATGTCCATCAGGTTCTTCCTGAC	transgene 238bp
CD11b-cre fwd	CGACCAGGTTCGTTCACTCA	
CD11b-cre rev	CAGCGTTTTCGTTCTGCCAA	transgene 184bp
Mpl fwd	CAGGAACCTGAGGGGCTGGC	
Mpl rev1	GTTTGGAAGGGCCAAGAGGA	WT allele 147bp
Mpl rev2	AAGCGCCATTCGCCATTCAG	knockout allele 205bp
LysM-cre fwd	GGGCTGCCAGAATTTCTC	
LysM-cre rev1	ACTCCATAGTAGCCAGCCATTCC	endogenous allele 350bp
LysM-cre rev2	GGTTATTCAACTTGCACCATGCC	transgene 450bp
Flt3-cre fwd	TCTGGGCCAAACCAAGTACCTGG	
Flt3-cre rev	CAAGACCAGACTGTGCAAAGGG	endogenous allele 332bp
Flt3-cre rev	GGGGACTTGCTCCCATGTAAA	transgene 950bp
**RT-PCR**		
FXIII-A fwd	TGCTGGTGTCTTTAACACATTTTTAA	
FXIII-A rev	TGGGCCGAGAATGAATTGGT	
β-actin fwd	CGTGAAAAGATGACCCAGATCA	
β-actin rev	TGGTACGACCAGAGGCATACAG	
RPL32 fwd	AAAATTAAGCGAAACTGGCGG	
RPL32 rev	TGTTGCTCCCATAACCGATG	
Pf4 fwd	CAGCTAAGATCTCCATCGCTTT	
Pf4 rev	AGTCCTGAGCTGCTGCTTCT	
CD11b fwd	AAACCACAGTCCCGCAGAGA	
CD11b rev	CGTGTTCACCAGCTGGCTTA	

References

1. Aydin IT, Tokcaer Z, Dalgic A, Konu O, Akcali KC. Cloning and expression profile of FLT3 gene during progenitor cell-dependent liver regeneration. *J Gastroenterol Hepatol.* 2007:22:2181-8.

2. Cordell PA, Kile BT, Standeven KF, Josefsson EC, Pease RJ, Grant PJ. Association of coagulation factor XIII-A with Golgi proteins within monocyte-macrophages: implications for subcellular trafficking and secretion. *Blood.* 2010:115:2674-81.
