# Supplementary figures and images for: Cre/lox Studies Identify Resident Macrophages as the Major Source of Circulating Coagulation Factor XIII-A
Source: Arterioscler Thromb Vasc Biol. 2017 Jul 26;37(8):1494–502. doi: 10.1161/ATVBAHA.117.309271 (PMC5526434; doi:10.1161/ATVBAHA.117.309271)

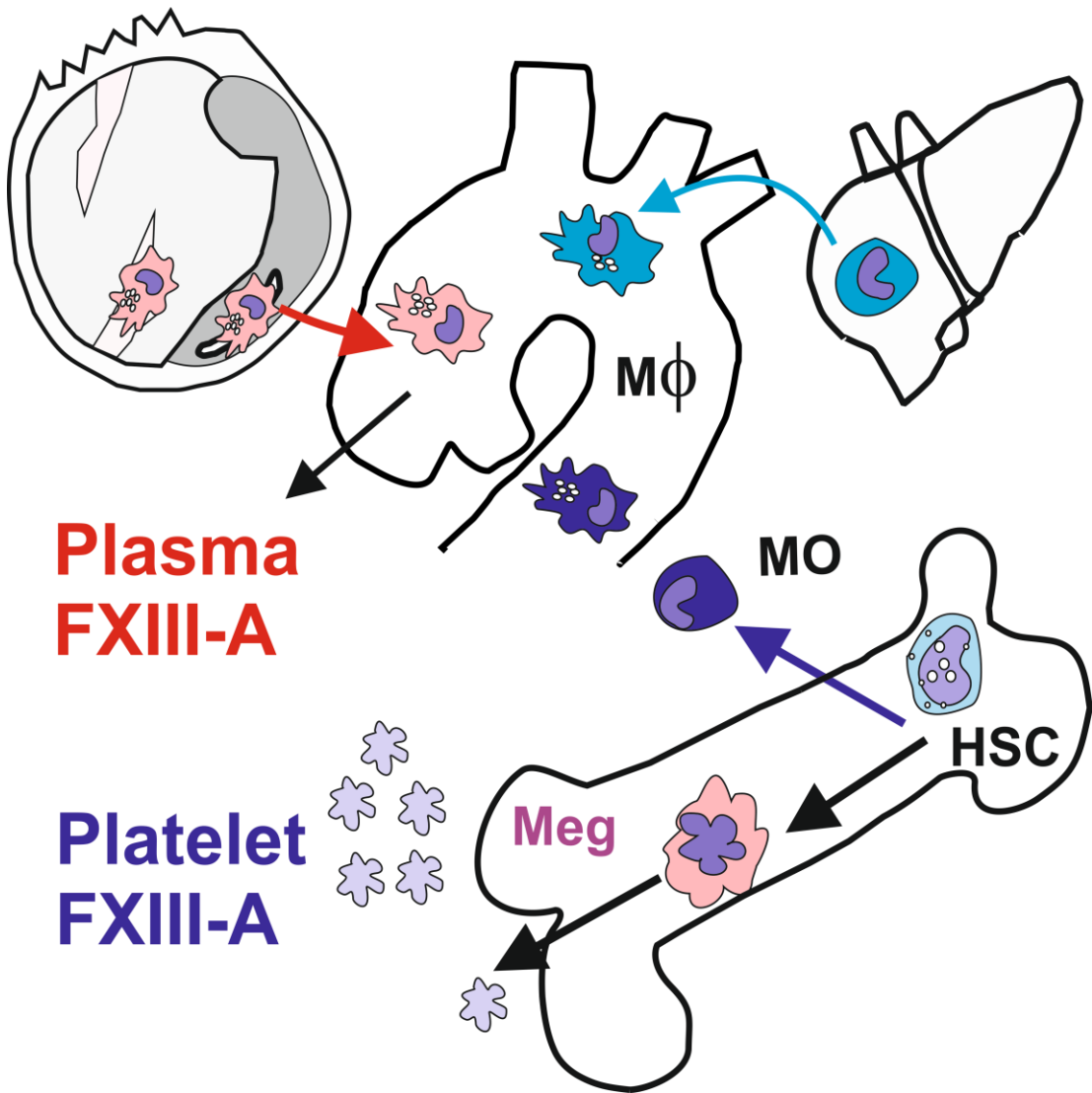

Supplement: Supplementary file 3 [file atv-37-1494-s003.pdf]
